# Development and Characterization of New Monoclonal Antibodies Against Porcine Interleukin-17A and Interferon-Gamma

**DOI:** 10.3389/fimmu.2022.786396

**Published:** 2022-02-03

**Authors:** Jean N. Manirarora, Kristen E. Walker, Veerupaxagouda Patil, Gourapura J. Renukaradhya, Joanna LaBresh, Yvonne Sullivan, Ore Francis, Joan K. Lunney

**Affiliations:** ^1^ Animal Parasitic Diseases Laboratory, Beltsville Agricultural Research Center (BARC), Agricultural Research Service (ARS), United States Department of Agriculture (USDA), Beltsville, MD, United States; ^2^ Center for Food Animal Health, Department of Animal Sciences, The Ohio State University, Wooster, OH, United States; ^3^ Kingfisher Biotech, Inc., St. Paul, MN, United States; ^4^ Bristol Veterinary School, University of Bristol, Bristol, United Kingdom

**Keywords:** swine, immunoassay, monoclonal antibodies, cytokines, interleukin-17A, interferon-gamma

## Abstract

Current research efforts require a broad range of immune reagents, but those available for pigs are limited. The goal of this study was to generate priority immune reagents for pigs and pipeline them for marketing. Our efforts were aimed at the expression of soluble swine cytokines and the production of panels of monoclonal antibodies (mAbs) to these proteins. Swine interleukin-17A (IL-17A) and Interferon-gamma (IFNγ) recombinant proteins were produced using yeast expression and used for monoclonal antibody (mAb) production resulting in panels of mAbs. We screened each mAb for cross-species reactivity with orthologs of IL-17A or IFNγ and checked each mAb for inhibition by other related mAbs, to assign mAb antigenic determinants. For porcine IL-17A, the characterization of a panel of 10 mAbs identified eight different antigenic determinants; interestingly, most of the mAbs cross-reacted with the dolphin recombinant ortholog. Likewise, the characterization of a panel of nine anti-PoIFNγ mAbs identified four different determinants; most of the mAbs cross-reacted with dolphin, bovine, and caprine recombinant orthologs. There was a unique reaction of one anti-PoIFNγ mAb that cross-reacted with the zebrafish recombinant ortholog. The αIL-17A mAbs were used to develop a quantitative sandwich ELISA detecting the yeast expressed protein as well as native IL-17A in stimulated peripheral blood mononuclear cell (PBMC) supernatants. Our analyses showed that phorbol myristate acetate/ionomycin stimulation of PBMC induced significant expression of IL-17A by CD3+ T cells as detected by several of our mAbs. These new mAbs expand opportunities for immunology research in swine.

## 1 Introduction

Immunological research in pigs remains hindered by limited reagent availability (1–4). Two cytokines of interest are interleukin-17A (IL-17A) and interferon-gamma (IFNγ). The IL-17 family is best known for its important role in host defense and immune pathology (5–7). Six members of the IL-17 family, annotated as IL-17A through IL-17F, have been identified (8, 9). IL-17A is an essential player in host disease defense; aberrant expression of IL-17A can lead to many autoimmune diseases and cancers. IL-17A signaling enhances production of proinflammatory molecules in multiple cell types. T helper 17 (Th17) cells (a subset of CD4+ T cells) and γδT cells are major producers of IL-17A although other cell subsets have been indicated (10).

The IFN family is best known for its important role in host immune response to infections, pathogens, and various diseases (11). IFNγ (the only type II IFN) is produced by many cell types, including, CD4+ T helper cell type 1 (Th1) lymphocytes, CD8+ cytotoxic lymphocytes, Natural Killer (NK) cells, NKT cells, and professional antigen-presenting cells: monocyte/macrophage, dendritic cells, and B cells. IFNγ plays a major role in the fight against viruses, intracellular bacteria, and tumors, and is generally anti-inflammatory in allergy and asthma (12). In pigs, IFNγ has been reported to play an important role in the remodeling of uterine endometrial epithelium and in promoting cell adherence during implantation (13).

As reported herein, we describe the development and characterization of panels of monoclonal antibodies (mAbs) to porcine IL-17A or IFNγ. The characterization of these new reagents includes antigen specificity, cross-clone inhibition, cross-species reactivity, intracellular staining in pig peripheral blood mononuclear cells (PBMCs), and successful development of a soluble protein detection assay.

## 2 Materials and Methods

### 2.1 Development and Characterization of Anti-Porcine IL-17A and Anti-Porcine IFNγ mAbs

Recombinant cytokine proteins were cloned and expressed in *Pichia pastoris* by Kingfisher Biotech, (Saint Paul, MN). At a contract facility, cytokine specific hybridomas were produced using BALB/c mice that were immunized subcutaneously twice at 4-week intervals (50 μg/dose) with swine IL-17A recombinant protein (rPoIL-17A; Kingfisher Biotech, Saint Paul, MN) or swine IFNγ recombinant protein (rPoIFNγ; Kingfisher Biotech, Saint Paul, MN). Once antibodies were detected in the serum, mice were injected with a final intravenous boost of rPoIL-17A or rPoIFNγ, and hybridoma fusion conducted (14). The primary hybridoma supernatants were screened for specificity by ELISA; supernatants positive for rPoIL-17A or rPoIFNγ, but negative for anti-carbohydrate reactivity, were cloned and expanded for mAb production and purification. A panel of 9 anti-PoIFNγ (αPoIFNγ) mAbs and 10 anti-PoIL-17A (αPoIL-17A) mAbs were selected for further characterization and validation by ELISA for specific binding, determinant reactivity, and intracellular staining (**Table 1**).

**Table 1:** Antibodies used in this study.

Antigen	Clone	Isotype	Format	Source
Porcine IL-17A	αPoIL-17A-1.1	IgG1	Pure, Biotin, AF647	Contract, In House
Porcine IL-17A	αPoIL-17A-1.2	IgG1	Pure, Biotin, AF647	Contract, In House
Porcine IL-17A	αPoIL-17A-2.1	IgG1	Pure, Biotin, AF647	Contract, In House
Porcine IL-17A	αPoIL-17A-2.3	IgG1	Pure, Biotin, AF647	Contract, In House
Porcine IL-17A	αPoIL-17A-2.4	IgG1	Pure, Biotin, AF647	Contract, In House
Porcine IL-17A	αPoIL-17A-2.5	IgG1	Pure, Biotin, AF647	Contract, In House
Porcine IL-17A	αPoIL-17A-2.6	IgG1	Pure, Biotin, AF647	Contract, In House
Porcine IL-17A	αPoIL-17A-2.8	IgG1	Pure, Biotin, AF647	Contract, In House
Porcine IL-17A	αPoIL-17A-2.9	IgG1	Pure, Biotin, AF647	Contract, In House
Porcine IL-17A	αPoIL-17A-2.10	IgG1	Pure, Biotin, AF647	Contract, In House
Cattle IL-17A	IL-17A2A	IgG	Pure, AF647	W Davis, WSU
Human IL-17A	SCPL1362	IgG1	AF647	BD Bioscience
Porcine IFNγ	αPoIFN-γ-1.1	IgG1	Pure, Biotin, AF647	Contract, In House
Porcine IFNγ	αPoIFN-γ-17.1	IgG1	Pure, Biotin, AF647	Contract, In House
Porcine IFNγ	αPoIFN-γ-21.3	IgG1	Pure, Biotin, AF647	Contract, In House
Porcine IFNγ	αPoIFN-γ-23.2	IgG1	Pure, Biotin, AF647	Contract, In House
Porcine IFNγ	αPoIFN-γ-24.1	IgG1	Pure, Biotin, AF647	Contract, In House
Porcine IFNγ	αPoIFN-γ-27.3	IgG1	Pure, Biotin, AF647	Contract, In House
Porcine IFNγ	αPoIFN-γ-34.2 -	IgG1	Pure, Biotin, AF647	Contract, In House
Porcine IFNγ	αPoIFN-γ-35.1 -	IgG1	Pure, Biotin, AF647	Contract, In House
Porcine IFNγ	αPoIFN-γ-45.2	IgG1	Pure, Biotin, AF647	Contract, In House
Porcine IFNγ	P2G10	IgG1	Pure, Biotin, AF647	BD Bioscience
Porcine CD3e	BB23-8E6-8C8	IgG2a	PE	BD Bioscience
Isotype Control	MOPC-21	IgG1	Pure, Biotin, AF647	BD Bioscience
Isotype Control	R35-95	IgG2a	PE	BD Bioscience

Shown is the list of antibodies used in this study, including original antigens, clone designations, IgG isotypes, formats, and sources.

### 2.2 ELISA Screening of Hybridoma Supernatants and mAb Characterization

Original hybridoma supernatants were screened by ELISA for antigen specificity with rPoIL-17A or rPoIFNγ, and for lack of reactivity with yeast carbohydrates using recombinant bovine IL-4 (rBoIL-4; Kingfisher Biotech, Saint Paul, MN). ELISA plates (ThermoFisher Fisher Scientific, Rochester, NY) were coated overnight with optimized amounts of recombinant proteins (1-2 μg/ml) in sodium carbonate/bicarbonate pH10 buffer. After washes, the plates were blocked with Phosphate Buffered Saline (PBS) with 1% Bovine Serum Albumin (BSA) (PBS-BSA) then washed before supernatants from different hybridoma clones were added for 1 hr. After washes, Peroxidase-Conjugated AffinePure Goat anti-mouse IgG (H+L) antibody (Jackson Immuno-Research Laboratories, West Grove, PA) was added and followed after 30 minutes with SureBlue Reserve Tetramethylbenzidine (TMB) Microwell Peroxidase Substrate (KPL, Gaithersburg, MD). Optical density (OD) at 650 nm was recorded using the VersaMax™ Tunable Microplate Reader (VWR, Radnor, PA). Only clones whose supernatants showed positive immunogen reactivity and no reactivity with rBoIL-4 protein (known high levels of carbohydrates) were selected for further expansion and screening for specificity.

### 2.3 Screening of mAbs for Specific Determinant Reactivity

Protein A purified αPoIL-17A or αPoIFNγ mAbs were biotinylated according to the manufacturer’s instructions using the EZ-Link™ Sulfo-NHS-LC-Biotin reagent (ThermoFisher Scientific, Waltham, MA). Purified αPoIL-17A or αPoIFNγ mAbs were incubated in ELISA plates precoated with purified rPoIL-17A or rPoIFNγ. The subsequent binding of biotin-labeled αPoIL-17A or αPoIFNγ mAbs was determined with Streptavidin-Horseradish Peroxidase conjugate (SAv-HRP) (Thermo Fisher Scientific, Waltham, MA). Percent inhibition of the binding of biotin-labeled mAb with a 100-fold excess of each purified (non-biotinylated) mAb was calculated. Antigenic determinants were assigned based on mAb cross-inhibition and binding to cross-species orthologs.

### 2.4 Screening of mAbs for Cross-Species Reactivity With Recombinant Orthologs

ELISAs were performed as noted in section *ELISA Screening of Hybridoma Supernatants and mAb Characterization*. Plates were coated with every available yeast expressed recombinant protein ortholog from different species (Kingfisher Biotech, Saint Paul, MN) and reactivity compared to that with rPoIL-17A or rPoIFNγ. Specifically, biotin-labeled anti-PoIL-17A mAbs were tested for binding to 13 rPoIL-17A orthologs from bovine, canine, mouse, dolphin, ovine, feline, zebrafish, human, caprine, rabbit, monkey, equine, and guinea pig; anti-PoIFNγ mAbs were tested against rPoIFNγ from the same species, except for guinea pig, which was replaced by murine. Any cross-species reaction whose ELISA OD was at equal or higher than 0.5 was considered positive.

### 2.5 Development of Sandwich ELISA for Quantitation of IL-17A

Sets of purified αPoIL-17A mAbs were tested to determine the optimal set of mAbs for quantitation of IL-17A. Capture mAbs were diluted in sodium carbonate buffer at optimal concentration (5 µg/µl) to coat ELISA plates (Thermo Fisher Scientific, Rochester, NY). Recombinant PoIL-17A (rPoIL-17A) (Kingfisher Biotech, Saint Paul, MN) was diluted serially from 0-100,000 pg/ml in PBS-BSA and added to wells for 1 hr to test for standard curve sensitivity. Biotinylated αPoIL-17A mAbs were added to each well at 0.1 µg/ml for 1 hr. After washing, SAv-HRP was added, and reactivity measured with SureBlue Reserve TMB Peroxidase Substrate. Once the best standard curves were established, reactivity with native PoIL-17A was tested using supernatants from stimulated peripheral blood mononuclear cells (PBMCs) and compared to media control.

### 2.6 PBMC Isolation and Stimulation for Cytokine Production

PBMCs were separated from pig blood by density centrifugation using a Lymphocyte Separation Medium LymphoSepTM (MP Biomedicals, Solon, OH) (15) and used fresh or frozen in liquid nitrogen until thawed for *in vitro* cultures. Frozen and fresh PBMC from multiple pigs were used for the studies conducted at BARC and OSU. All cell cultures were conducted in blastogenic medium [850 ml RPMI 1640 medium (ThermoFisher Scientific, Waltham, MA), 100 ml fetal bovine serum (FBS) (HyClone, Logan, UT), 25 ml 1M HEPES pH7.3, 5 ml 2-mercaptoethanol, 10 ml (10000UI/10000µg) Penicillin-Streptomycin, 10 ml 200 mM L-glutamine]. All cultures were incubated at 37°C/5% CO_2_.

Native PoIL-17A was prepared from PBMC cultured in 6 well plates at 4 x 10^6^ cells/well. Cells were cultured in blastogenic medium with phytohemagglutinin (PHA) at 10 μg/ml or phorbol myristate acetate and ionomycin (PMA/Iono) at 50 ng/ml and 500 ng/ml, respectively, to induce cytokine production. Cells were incubated for 24 or 48 hrs in a 37°C humidified CO_2_ incubator, then harvested and centrifuged. Supernatants were collected, aliquoted, and stored at -20°C until use for ELISA assay and not refrozen after use.

### 2.7 Cell Culture for Immunostaining and Flow Cytometric Analyses

Purified αPoIL-17A or αPoIFNγ mAbs were labeled with AF647 according to the manufacturer’s instructions using the Alexa Fluor^®^ 647 Protein Labeling Kit (ThermoFisher Scientific, Waltham, MA). For cell culture, frozen PBMCs were thawed and cultured overnight in blastogenic medium in 6 well plates at 4 x 10^6^ cells/well. Cells were stimulated for 5 hrs with BD Leukocyte Activation Cocktail (BD Biosciences, San Diego, CA), which is a ready-to-use polyclonal cell activation mixture containing PMA/Iono, and a protein transport inhibitor (Brefeldin A). For cell surface staining, Fc receptors were first blocked for 30 min at 4°C in complete Flow Cytometry Medium (FCM) [PBS-BSA with 20 mM of Sodium Azide, and 1% normal Rabbit Serum]. For dead/viable cell exclusion, the cells were stained with the fixable viability stain 520 (FVS) (BD Biosciences, San Diego, CA) in PBS for 7 min at 37°C and washed twice with PBS-BSA. Alternatively, the cells were stained with the VivaFix cell viability dye (Bio-Rad, Hercules, CA) in PBS for 30 min at room temperature, then washed twice with PBS-BSA.

For intracellular staining, after staining for dead/viable cell exclusion as noted above, Fc receptors were blocked for 30 min at 4°C in complete FCM. The cells were then stained with PE-conjugated αPoCD3 mAb (BD Biosciences, San Diego, CA) for 30 min at 4°C in normal FCM, fixed for 30 min at 4°C in Fixation & Permeabilization Buffer (BD Biosciences, San Diego, CA), washed twice with 1x Permeabilization Buffer (BD Biosciences, San Diego, CA), and labeled with AF647-conjugated αPoIL-17A or αPoIFNγ mAb (**Table 1**) for 30 min at 4°C in 1x Permeabilization Buffer. The cells were washed twice with 1x Permeabilization Buffer and re-suspended in normal FCM. For flow cytometric analyses, data on labeled cells were acquired either on an Accuri C6 or an Accuri C6 Plus flow cytometer (BD Biosciences, San Diego, CA) and analyzed using FlowJo Software, version 10.7.1 (BD Biosciences, San Jose, CA), gating on live lymphocytes and live T cells.

### 2.8 BLAST and Multiple Sequence Alignments

Single best sequence homologs for each immunogen were retrieved from NCBI protein BLAST searches (https://blast.ncbi.nlm.nih.gov/Blast.cgi), performed restricting hits to a single sequence and using all other default settings. Corresponding swine reference amino acid sequences were BLAST queries. Hits corresponding to species of interest were aligned using the NCBI COBALT tool (https://www.ncbi.nlm.nih.gov/tools/cobalt/cobalt.cgi) with default settings.

### 2.9 Data Analysis

ELISA data were analyzed using Microsoft Excel program (Microsoft Software, Redmond, WA). The mean ODs of duplicates were plotted using GraphPad Prism 5 software (GraphPad Software, La Jolla, CA). Any cross-species reaction whose optical density (OD) was equal to, or higher than, 0.5 was considered positive. The lower and upper sensitivity limits of the sandwich ELISA were determined as the first data point which detects reactivity above background level, and the upper sensitivity limit was determined as the apex point on the standard curve. Flow Cytometry data were analyzed using FlowJo Software version 10.7.1 (BD Biosciences, San Jose, CA), gating on live lymphocytes and for some analyses on live CD3+ T cells.

## 3 Results

### 3.1 Characterization of Anti-IL-17A mAb Antigenic Determinants

To define individual antigenic determinants that are recognized by each of the 10 αPoIL-17A mAbs, a competition ELISA was used to measure the ability of excess unlabeled mAb to inhibit the binding of each biotin-labeled mAb to the target rPoIL-17A. Shown in **Table 1** is the complete list of antibodies used in these studies. **Table 2** notes percent inhibition of the binding of biotin-labeled mAbs by a 100-fold excess of the unlabeled mAbs. Several biotin-labeled mAbs (αPoIL-17A-2.4, -2.5, -2.6, and -2.8) were inhibited by self and most other αPoIL-17A mAbs, whereas a few others (αPoIL-17A-1.1, -1.2) were inhibited by no or a few other mAbs.

**Table 2:** Determinant analyses of anti-PoIL-17A mAbs.

Non-Bio	Bio									
IL-17A mAbs	IL-17A-1.1	IL-17A-1.2	IL-17A-2.1	IL-17A-2.3	IL-17A-2.4	IL-17A-2.5	IL-17A-2.6	IL-17A-2.8	IL-17A-2-9	IL-17A-2.10
**IL-17A-1.1**	**38.7**	**30.8**	**56.6**	**44.0**	**67.1**	**44.0**	**67.4**	**43.6**	**71.8**	**56.9**
**IL-17A-1.2**	**10.3**	**34.8**	**61.2**	**54.2**	**85.4**	**59.0**	**84.9**	**82.7**	**65.5**	**56.1**
**IL-17A-2.1**	**0.0**	**0.0**	**48.9**	**54.2**	**59.3**	**55.3**	**58.8**	**57.8**	**38.1**	**36.1**
**IL-17A-2.3**	**0.0**	**0.0**	**44.2**	**47.8**	**55.3**	**56.5**	**54.7**	**56.9**	**27.5**	**39.7**
**IL-17A-2.4**	**0.0**	**0.0**	**35.6**	**17.7**	**39.9**	**38.5**	**39.8**	**47.8**	**19.4**	**28.6**
**IL-17A-2.5**	**26.5**	**36.3**	**67.8**	**75.4**	**88.5**	**74.0**	**88.0**	**69.2**	**49.4**	**58.7**
**IL-17A-2.6**	**0.0**	**10.5**	**58.5**	**20.7**	**70.6**	**57.4**	**67.9**	**64.0**	**31.3**	**33.8**
**IL-17A-2.8**	**0.0**	**0.0**	**31.6**	**20.7**	**39.5**	**29.8**	**41.5**	**45.9**	**36.7**	**29.3**
**IL-17A-2-9**	**0.0**	**0.0**	**31.7**	**20.2**	**37.8**	**28.9**	**39.8**	**51.2**	**35.0**	**31.2**
**IL-17A-2.10**	**0.0**	**9.5**	**63.8**	**64.3**	**74.5**	**59.7**	**69.7**	**75.5**	**36.8**	**50.4**
**Cross-species reactivity**	**Dolphin, Human, Monkey**	**None**	**Dolphin, Human, Equine, Guinea Pig, Bovine**	**Dolphin, Human, Equine, Guinea Pig, Bovine**	**Dolphin, Human, Monkey, Equine, Guinea Pig, Bovine**	**None**	**Dolphin, Human, Equine, Guinea Pig, Bovine**	**Dolphin, Human, Guinea Pig**	**Monkey**	**Dolphin, Human, Monkey, Equine, Caprine, Ovine, Bovine, Rabbit**
**Determinants**	**A**	**B**	**C**	**C**	**D**	**E**	**C**	**F**	**G**	**H**
**Cross-clones inhibition**	**>60%**	**30-60%**	**<30%**	**Self**						

Shown are percent inhibition of the binding of biotinylated anti-PoIL17A mAbs by a 100-fold excess of the unlabeled anti-PoIL-17A mAbs. Numbers in yellow reflect self-inhibition, in orange >60% inhibition, gray 30-60% inhibition, and blue <30% inhibition. The cross-species reactivities summarized and assigned determinant groups noted.

### 3.2 Cross-Reactivity of Anti-IL-17A mAbs With Orthologous Recombinant Proteins and Determinant Assignments

**Figure 1:**
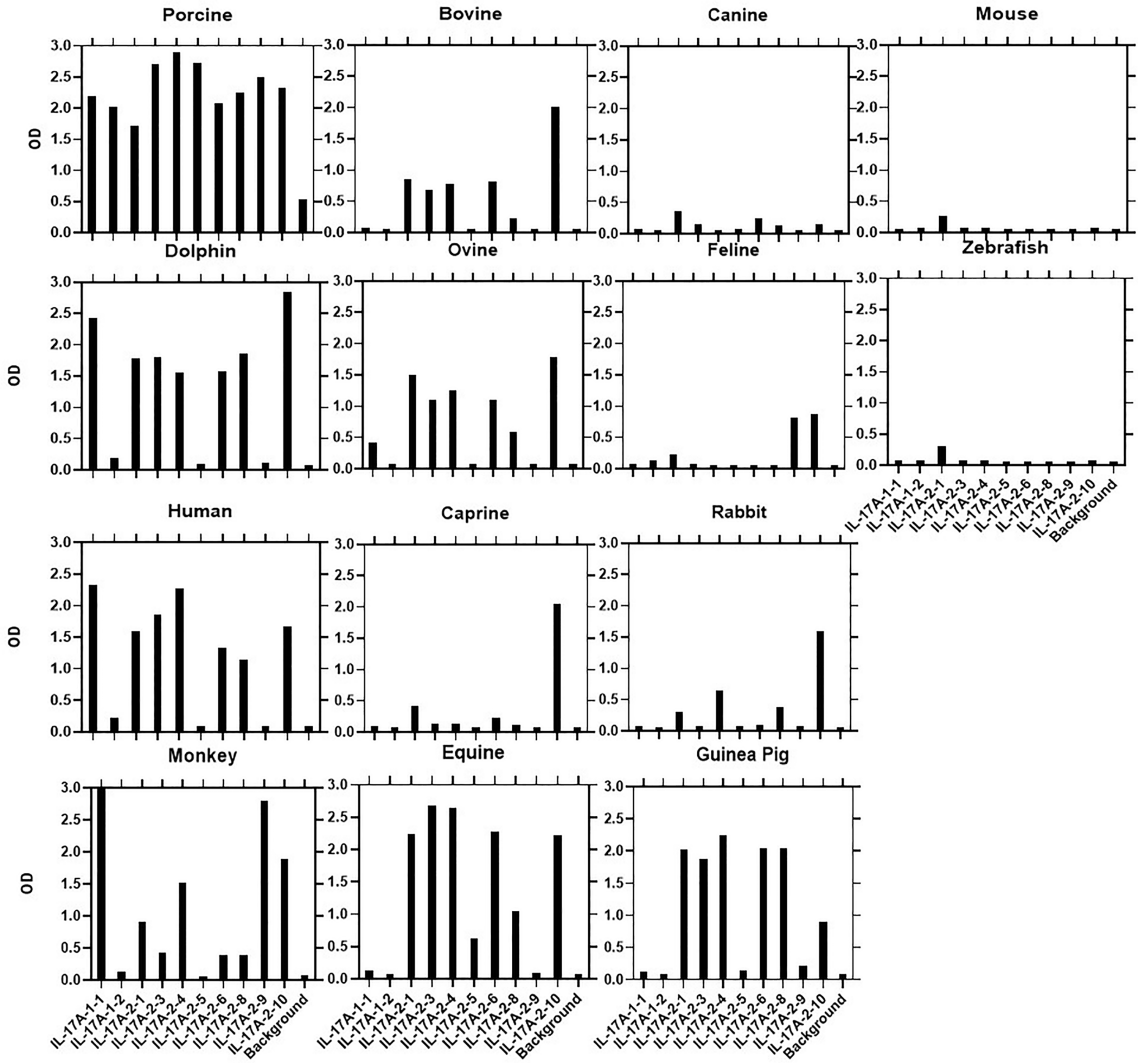
Cross-species binding of αPoIL-17A mAbs. A panel of biotin-labeled αPoIL-17A mAbs were tested for their ability to bind to purified orthologous rIL-17A proteins from species as described in M&M. Shown are mean ODs of duplicates for the binding of 1μg/ml for each mAb.

Most αPoIL-17A mAbs showed varying levels of cross-reactivity with orthologous rIL-17A proteins (**Figure 1**). Only one mAb (αPoIL-17A-1.2) did not cross-react with any orthologs we tested. Based on the cross-reactivity patterns shown in **Figure 1**, and the inhibition patterns shown in **Table 2**, eight antigenic determinants (A-H) were proposed for the 10 αPoIL-17A mAbs: Group A: Represented by αPoIL-17A-1.1, this mAb cross-reacted with human, dolphin, and monkey rIL-17A proteins and was not inhibited by any of the other mAbs. Group B: Represented by αPoIL-17A-1.2, this mAb did not cross-react with any orthologous rIL-17A proteins we tested. Group C: Represented by αPoIL-17A-2.1, -2.3, and -2.6, these mAbs cross-reacted with human, dolphin, equine, guinea pig, bovine, and ovine rIL-17A and had similar cross-inhibition patterns. Group D: Represented by αPoIL-17A-2.4, this mAb cross-reacted with human, dolphin, monkey, equine, guinea pig, bovine and ovine rIL-17A. Group E: Represented by αPoIL-17A-2.5, this mAb cross-reacted with only equine IL-17A. Group F: Represented by αPoIL-17A-2.8, this mAb cross-reacted with dolphin, guinea pig, human, equine, and ovine rIL-17A. Group G: Represented by αPoIL-17A-2.9, this mAb cross-reacted with monkey and weakly with feline rIL-17A. Group H: Represented by αPoIL-17A-2.10, this mAb cross-reacted with dolphin, human, monkey, equine, caprine, ovine, bovine, rabbit, guinea pig, and weakly with feline rIL-17A. No cross-reactivity was found for any αPoIL-17A mAb with canine, mouse, or zebrafish rIL-17A.

### 3.3 Sequence Alignments for Porcine IL-17A Proteins

We performed a BLAST search on porcine IL-17A proteins to see whether amino-acid sequences that are shared with several orthologous proteins we tested, may correlate with the cross-species reactivity patterns we observed in this study (**Supplementary Figure 1**). Next to each sequence name is a percentage identity value with respect to the aligned region. For porcine IL-17A, the closest ortholog is bovine (83% identity), then ovine, caprine, and dolphin, all of which with 81% identity. Despite the highest sequence identity mAb reactivity with the rBoIL-17A ortholog was relatively low; higher reactivity was found with equine and human rIL-17A with 74% and 72% sequence identity, respectively, to porcine IL-17A.

### 3.4 Characterization of Anti-IFNγ mAb Antigenic Determinants

As with IL-17A, porcine IFNγ antigenic determinants recognized by each of the nine αPoIFNγ mAbs were defined by competition ELISA, testing 100-fold excess unlabeled αPoIFNγ mAbs capacity to competitively inhibit binding of biotin-labeled αPoIFNγ mAbs to rPoIFNγ. As shown in **Table 3**, for αPoIFNγ mAbs, half of the unlabeled αPoIFNγ mAbs inhibited the binding of self biotin-labeled mAbs (αPoIFNγ-1.1, -17.1, -21.3, -23.2). The other inhibition patterns were heterogeneous, ranging from none, weak to medium, or strong. There were also some mAbs that failed to inhibit self (αPoIFNγ-35.1 and -45.2) (**Table 3**).

**Table 3:** Assignment of anti-PoIFNγ mAbs determinants.

Non-bio	Bio								
IFNγ mAbs	IFNγ-1.1	IFNγ-17.1	IFNγ-21.3	IFNγ-23.2	IFNγ-24.1	IFNγ-27.3	IFNγ-34.2	IFNγ-35.1	IFNγ-45.2
**IFNγ-1.1**	**60.7**	**80.1**	**59.2**	**12.5**	**35.6**	**NT**	**2.8**	**45.1**	**0.0**
**IFNγ-17.1**	**30.4**	**74.9**	**97.0**	**43.0**	**59.4**	**NT**	**58.8**	**26.7**	**27.7**
**IFNγ-21.3**	**35.5**	**100.0**	**100.0**	**54.5**	**68.2**	**NT**	**77.0**	**55.4**	**57.5**
**IFNγ-23.2**	**49.5**	**92.4**	**79.6**	**67.9**	**80.1**	**NT**	**37.6**	**51.7**	**42.2**
**IFNγ-24.1**	**34.4**	**30.1**	**53.4**	**0.0**	**27.3**	**NT**	**0.0**	**47.1**	**0.0**
**IFNγ-27.3**	**13.0**	**50.6**	**89.0**	**0.0**	**37.9**	**NT**	**12.8**	**13.1**	**20.0**
**IFNγ-34.2**	**17.2**	**44.8**	**47.8**	**0.0**	**31.0**	**NT**	**23.3**	**0.0**	**6.2**
**IFNγ-35.1**	**15.0**	**67.7**	**58.3**	**0.0**	**52.8**	**NT**	**15.2**	**0.0**	**35.4**
**IFNγ-45.2**	**36.3**	**0.0**	**70.3**	**0.0**	**0.0**	**NT**	**45.8**	**72.6**	**0.0**
** **									
**Cross-species reactivity**	**Zebrafish**	**Dolphin, Bovine, Canine, Caprine, Ovine, Feline**	**Dolphin, Bovine, Canine, Caprine, Ovine**	**Dolphin, Bovine, Caprine**	**Dolphin, Bovine, Caprine, Human, Equine, Ovine**	**Dolphin, Bovine, Canine, Caprine, Ovine, Feline**	**Dolphin, Bovine, Canine, Caprine, Ovine, Feline**	**Dolphin, Bovine, Caprine,**	**Dolphin, Bovine, Caprine, Canine, Ovine, Feline, Equine**
**Determinants**	**A**	**C**	**C**	**B**	**(B)**	**D**	**D**	**(B)**	**D**
**Cross-clones inhibition**	**>60%**	**30-60%**	**<30%**	**Self**					

Shown are percent inhibition of the binding of biotinylated anti-PoIFNγ mAbs by a 100-fold excess of the unlabeled anti-PoIFNγ mAbs. Numbers in yellow reflect self inhibition, in orange >60% inhibition, gray 30-60% inhibition, and blue <30% inhibition. The cross-species reactivities summarized and assigned determinant groups noted. NT: Not Tested.

### 3.5 Cross-Reactivity of Anti-IFNγ mAbs With Orthologous Recombinant Proteins and Determinant Assignments

**Figure 2:**
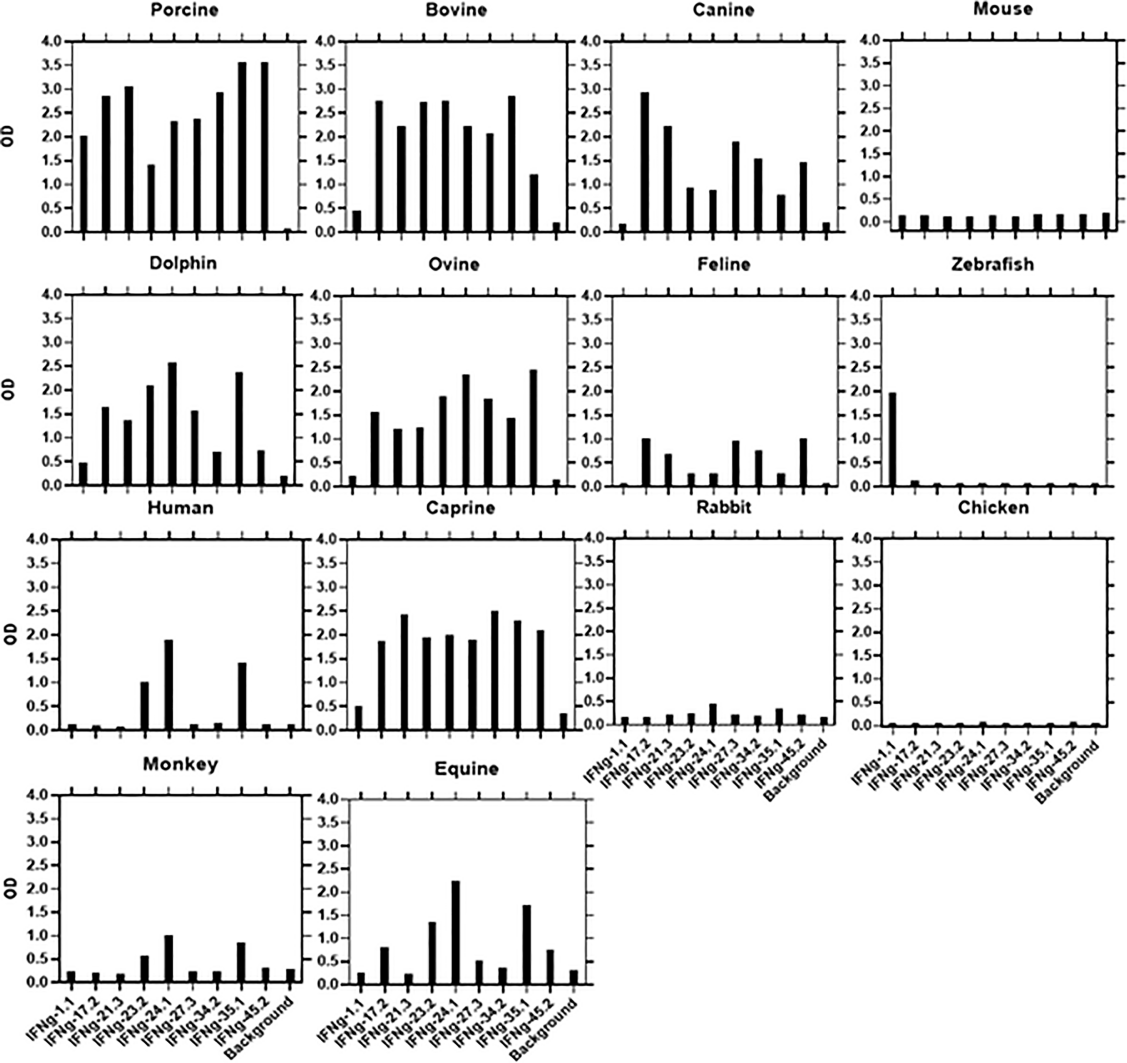
Cross-species binding of αPoIFNγ mAbs. A panel of biotin-labeled αPoIFNγ mAbs were tested for their ability to bind purified orthologous rIFNγ proteins from several species as described in M&M. Shown are mean ODs of duplicates for the binding of 1μg/ml for each mAb.

We used a direct ELISA to assess the ability of the αPoIFNγ mAbs to react with orthologous rIFNγ proteins. Fourteen orthologous rIFNγ proteins from different species were tested. Based on the binding patterns (**Figure 2**), four determinants (A-E) representing potential antigenic determinants emerged (**Table 3**). For group A, one mAb (αPoIFNγ-1.1) was the only mAb that unexpectedly cross-reacted with zebrafish rIFNγ protein. Most of the αPoIFNγ mAbs showed a strong cross-reactivity with orthologous rIFNγ proteins from dolphin, bovine, caprine, and ovine. Three αPoIFNγ mAbs (αPoIFNγ-23.2, -24.1, -35.1) also cross-reacted weakly with human and equine rIFNγ and were assigned as Group B. Group C: Represented by αPoIFNγ-17.1 and -21.3, these mAbs cross-reacted with canine, dolphin, bovine, ovine and caprine rIFNγ and had strong cross inhibition patterns. Group D: Represented by αPoIFNγ-27.3; -34.2 and -45.2, these mAbs cross-reacted with bovine, ovine, caprine, dolphin, and canine rIFNγ. No αPoIFNγ mAb cross-reactivity was detected with rabbit, chicken, or mouse rIFNγ, and only weak reactivity was seen with feline and monkey rIFNγ.

### 3.6 Sequence Alignments for Porcine IFNγ Proteins

Shown in **Supplemental Figure 2** are multiple sequence alignments against porcine IFNγ. Next to each sequence name is a percentage identity value with respect to the aligned region. For porcine IFNγ, the closest sequence homologies are with dolphin (80.7% identity), then bovine, ovine, and caprine, all of which with 77-78% identity. This sequence homology may explain why most of our new mAbs cross-reacted with bovine, ovine, caprine, equine, canine, feline, dolphin, and human IFNγ. However, it cannot explain the unique cross-reactivity of one mAb, αPoIFNγ-1.1, with zebrafish. Indeed, zebrafish IFNγ protein was the least identical to porcine IFNγ protein, with only 25% identity.

### 3.7 Sandwich ELISA and Quantitation of IL-17A

**Figure 3:**
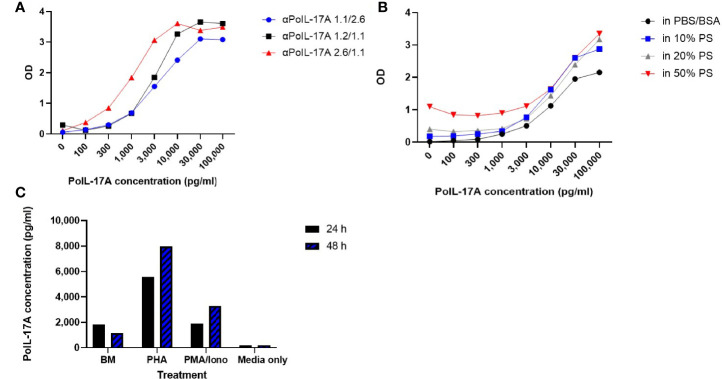
Sandwich ELISA Assay and Quantitation of IL-17A. **(A)** Comparison of IL-17A standard curves generated with 3 sets of αPoIL-17A mAb pairs as capture vs detection respectively: αPoIL-17A-1.1/2.6; 1.2/1.1; and 2.6/1.1; **(B)** Detection of rPoIL-17A diluted in PBS-BSA or in pig serum using the anti-PoIL-17A-1.1/2.6 mAb Sandwich assay. The assays were repeated >5 times; **(C)** Detection of native porcine IL-17A in PBMC supernatants using the anti-PoIL-17A-1.1/2.6 mAb assay. Supernatants were harvested from cells cultured for 24 or 48 hours (24 h/48 h) in medium (BM), PHA or PMA/Iono stimulated cells as described in M&M. This assay was conducted >3 times evidencing repeatability.

We established an IL-17A sandwich ELISA using rPoIL-17A standard curves and testing several sets of mAbs based on their different determinant reactivities. As shown in **Figure 3A**, three of the best performing mAb pairs were compared with αPoIL-17A-2.6 mAb as the capture mAb, and αPoIL-17A-1.1 mAb as the detection mAb (2.6/1.1 pair) proving to be the most sensitive pair at detecting rPoIL-17A with a lower sensitivity limit of 100-300 pg/ml and an upper limit of 3,000 pg/ml. The reverse mAb pair (1.1/2.6) and a different pair (1.2/1/1) were less sensitive with a lower limit of 300 pg/ml and an upper limit of 10,000 pg/ml. We did not test our newly developed anti-IFNγ mAbs for development of a sandwich ELISA because such assays are already commercially available from both R&D Systems and BD Biosciences.

Subsequent experiments were performed using the αPoIL-17A-2.6/-1.1 pair. We investigated the ELISA sensitivity for rPoIL-17A detection in PBS+BSA versus pig serum. As shown in **Figure 3B**, the lower sensitivity limit for the PBS+BSA curve was at 300 pg/ml, while the lower limit was around 1,000 pg/ml in the samples containing pig serum at 10% and 20%. There was an increasingly higher background with increasing concentrations of pig serum. This may make it more difficult to use this assay with samples containing >20% pig serum, because the PBS+BSA standard curve had better background levels. Thus, it is recommended to use samples containing no more than 20% pig serum to maintain the sensitivity of the assay.

Next, we needed to affirm the reactivity of the assay with native porcine IL-17A. As shown in **Figure 3C**, the αPoIL-17A-2.6/1.1 mAb pair was sensitive for detecting IL-17A in supernatants from cultured pig PBMC. This was evidenced by the low background levels in the cell culture medium only and the supernatant from unstimulated cells (BM) compared to the detection of native porcine IL-17A in supernatants from PHA or PMA/Iono-stimulated PBMC. More IL-17A was expressed in supernatants from PBMC stimulated with PHA or PMA/Iono for 48 hrs compared to supernatants from PBMC stimulated with PHA or PMA/Iono for 24 hrs.

### 3.8 Intracellular Staining of Pig PBMCs With AF647 Labeled Anti-PoIL-17A mAbs

**Figure 4:**
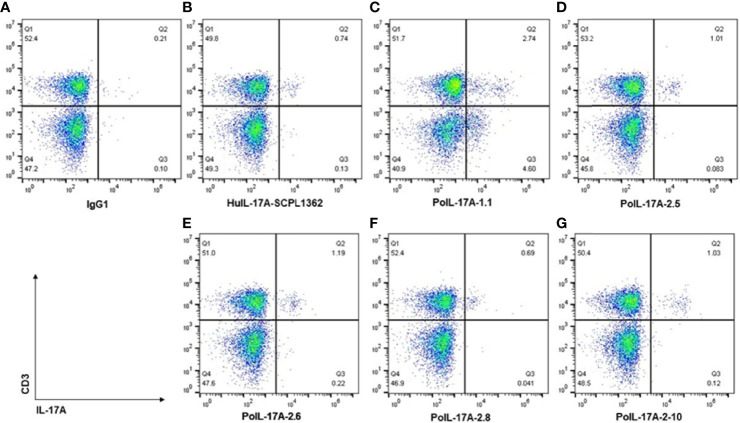
Intracellular staining of pig cells with AF647 labeled αPoIL-17A mAbs. Frozen PBMC were cultured overnight before stimulation with BD Leukocytes activation cocktail [containing phorbol myristate acetate (PMA)/ionomycin), and a protein transport inhibitor (Brefeldin A)]. Cells were stained with BD fixable viability stain and Fc receptors were blocked with rabbit serum before surface staining with αCD3 mAb. Cells were then fixed and permeabilized before intracellular staining with each AF647 labeled αPoIL17A mAb. Data were collected using flow cytometry, gating on live lymphocytes and on live CD3+ T cells, and analyzed using FlowJo Software. Shown are staining data for controls **(A, B)** and 5 new anti-PoIL-17A mAbs **(C–G)**.

We tested the ability of newly produced mAbs to specifically bind porcine IL-17A intracellularly. Several of the tested αPoIL-17A mAbs stained well and our analyses showed that PMA/ionomycin stimulation induced significant expression of IL-17A by CD3+ T cells. As shown in **Figure 4**, gating on live lymphocytes, the tested αPoIL-17A mAbs showed a clear population of T cells expressing IL-17A. Repeated assays revealed αPoIL-17A-2.5, -2.6, and -2.10 to be the most reliable mAbs. The mean frequency of IL-17A expressing CD3+ T cells from PBMCs was 1.33 ± 0.36%. Using αHuIL-17-SCPL1362 mAb as a positive control, our data confirmed a previous report that it cross-reacted with pig cells, staining 0.64 ± 0.19% CD3+ T cells after PMA/Iono stimulation (16). As shown in **Figure 4** three of our mAbs, αPoIL-17A-2.5, -2.6, and -2.10 stained 1.01, 1.19 and 1.03% PMA/ionomycin stimulated cells, respectively. One mAb, αPoIL-17A-2.8, only stained 0.69% of those same cells. We also compared our mAbs with αBoIL-17A-2A mAb, which we confirmed was cross-reactive with swine IL-17A (3) (data are not shown).

### 3.9 Intracellular Staining of Pig PBMCs With AF647-Labeled Anti-PoIFNγ mAbs

Four mAbs (αPoIFNγ-1.1, 17.1, 34.2, and 35.1) labeled with AF647 were compared to αPoIFNγ P2G10 (BD Bioscience, San Diego, CA) labeled with AF647 for their ability to bind to native IFNγ in PBMC stimulated for 5 hrs with BD leukocytes activation cocktail. With the gate set on live lymphocytes and CD3+ T cells, we found unusually high frequencies of IFNγ positive cells with one mAb (anti-PoIFNγ-1.1; **Supplementary Figure 3C**), which were not plausible, likely due to cell shift upon staining with the AF647-conjugated mAbs. Overall, none of the tested new anti-IFNγ mAbs worked in intracellular staining (**Supplementary Figure 3**).

## 4 Discussion

We report herein, the establishment of new panels of hybridomas that secrete αPoIL-17A and αPoIFNγ mAbs. The binding specificity of these mAbs has been characterized by direct ELISA, sandwich ELISA, and flow cytometry. IL-17A is an ideal drug target because of its broad involvement in many host defense mechanisms by inducing proinflammatory cytokines and chemokines that participate in neutrophil and macrophage recruitment to the site of injury (17). Indeed, recent reports have shown that IL-17A plays a role in skin wound healing (18) and gut epithelial repair (19) while αIL-17A and αIFNγ mAbs have been developed to treat psoriasis (20) and lymphohistiocytosis (21), respectively, in humans, with favorable outcomes. IL- 17A and IFNγ are therefore ideal targets for the future development of therapeutic antibodies to test using the pig biomedical model, to affirm the cytokine’s involvement in many host-defense mechanisms (21–23).

All mAbs showed a strong binding specificity to their target porcine antigen and various levels of cross-clone inhibition. Additionally, mAbs yielded several binding patterns when tested on orthologous cytokines, suggesting binding of different antigenic determinants on the corresponding porcine target. The fact that there were some αPoIFNγ mAbs that failed to inhibit self (**Table 3**), suggests that these mAbs may react with different determinants on rPoIFNγ. Highly specific mAbs often have varying potencies against antigen orthologs, which can affect the efficacy of these molecules in different animal models of disease (24). Usually, mAbs bind non-linear epitopes that depend on the precise three-dimensional arrangement of a constellation of amino acids. For this reason, mAbs raised against a specific antigen from any given species often do not cross-react with orthologs of that antigen from other species, and when they do, the binding potency may vary depending on the origin of the antigen that was used to test the mAbs. We found similar variations in this study when we tested the cross-reactivity of our new mAbs against 13 species orthologs. Some αPoIL-17A mAbs reacted strongly with multiple mammalian species, such as αPoIL-17A determinant H (**Table 2**), others showed no cross-species reactions, such as αPoIL-17A determinant B & E (**Table 2**). Still other mAbs showed unique reactions, such as αPoIFNγ determinant A reactivity on zebrafish rIFNγ (**Table 3**). Some cross inhibitions were strong such as αPoIFNγ determinant C where αPoIFNγ-17.1 and αPoIFNγ-21.3 fully cross inhibited each other but yet had very different inhibition by αPoIFNγ-45.2 (**Table 3**). The assignment of antigen determinant groups was not an easy task. Even though determinants were assigned based on mAb cross-clones inhibition and binding to cross-species orthologs, some were heterogeneous within the same determinant group (**Tables 2**, **3**). Additional studies are needed to elucidate whether these mAb antigenic determinants represent true epitopes. The cross-reaction of these mAbs with orthologous cytokines may provide needed reagents for several other species. Further analyses are needed to determine whether some of these mAbs are neutralizing, i.e., block IL-17A or IFNγ binding to the target receptor.

The key to the success of a sandwich ELISA is to identify an appropriate pair of capture and detection antibodies. Such an antibody pair should be highly specific and sensitive to accurately quantify a low level of antigens (pg/ml). We noted that there was higher background with increasing concentrations of pig serum, preventing optimal PoIL-17A detection, but good sensitivity with samples from cell supernatants. It may be more difficult to use this assay with samples containing higher concentrations of pig serum (higher than 10-20% pig serum). Notably, our newly developed αPoIL17A sandwich ELISA successfully detected native PoIL-17A in PBMC supernatants. The highest level of IL-17A was detected in PBMCs stimulated with the mitogen PHA after 48 hrs (**Figure 3C**). These data show that PHA was more successful in initiating cytokine IL-17A release from porcine PBMC than PMA/Iono, and that with increased stimulant incubation time, more cytokine is released with both stimulants. We did not test our newly developed anti-IFNγ mAbs in a sandwich ELISA because this assay is already commercially available.

For the tested αPoIL-17A mAbs, we describe a robust IL-17A flow cytometry-based assay to quantify and phenotype IL-17A-specific CD3+ T cells *ex-vivo* using cryopreserved PBMCs. Our intracellular staining data for PoIL-17A affirmed that several of our new αPoIL-17A mAbs appeared to be slightly better than the cross reactive αHuIL-17A-SCPL1362 mAb at detecting CD3+IL-17A+ cells, indicating that the anti-porcine mAbs may detect a broader range of IL-17A+ cells. Panels of these mAbs are being shared with colleagues to affirm whether this difference is found *in vivo*. MAbs are being shared *via* Material Transfer Agreements (MTAs; mAb commercialization is underway with our Technology Transfer Office.

The availability of these mAbs that detect cytokines by ELISA and flow cytometry will help dissect host response to pathogens and vaccines in swine. Comparative flow cytometric analyses of lymphocyte subsets may reveal differences in expression of IL-17A by CD4, CD8, and γδ T cells in swine (3). In addition, some of our mAbs may be useful for other species because they cross-reacted with their recombinant ortholog antigens. More studies are needed to elucidate this issue.

In conclusion, we have developed and characterized new panels of αPoIL-17A and αPoIFNγ mAbs. These mAbs provide the research community with new tools for the characterization of IL-17A-producing cells in pigs and the quantification of secreted IL-17A *in vitro*.

## Data Availability Statement

The original contributions presented in the study are included in the article/**Supplementary Material**. Further inquiries can be directed to the corresponding author.

## Author Contributions

JLu, JLa, GR conceptualized the study and secured funding for the project. JL and YS provided the cloned cytokines. JM, KW, VP, and OF conducted the experiments and performed the data analyses. All authors assisted in the review of the manuscript and consented to publication. All authors read and approved the final manuscript.

## Funding

This work was supported by funding from USDA ARS (project 8042-32000-102) and USDA NIFA Toolkit grants (2015-67015-23216; 2019-67015-29815).

## Conflict of Interest

JLa and YS are employed by Kingfisher Biotech, Inc., St. Paul, MN, USA.

The remaining authors declare that the research was conducted in the absence of any commercial or financial relationships that could be construed as a potential conflict of interest.

The reviewer SW has declared a past co-authorship with one of the author JL to the handling editor at the time of review.

## Publisher’s Note

All claims expressed in this article are solely those of the authors and do not necessarily represent those of their affiliated organizations, or those of the publisher, the editors and the reviewers. Any product that may be evaluated in this article, or claim that may be made by its manufacturer, is not guaranteed or endorsed by the publisher.

## Supplementary Material

Supplementary Figure 1 Sequence alignments for porcine IL-17A protein. Species orthologs of IL-17A were retrieved from the non-redundant databases of the National Center for Biotechnology Information (NCBI) using BLASTp searches with the corresponding porcine reference amino acid sequences (NP_001005729.1). Retrieved sequences were aligned using the NCBI constraint-based alignment tool (COBALT). Alignment outputs, percentage identity labels and (default) consensus lines for 100%, 90%, 80% and 70% thresholds were generated using the MView tool. Consensus shading was applied using the “any” parameter. Upper case characters (amino acid residues) are shaded using the default CLUSTAL colormap. Lower case characters in consensus lines correspond to physicochemical amino acid classes (25) also available at https://vcru.wisc.edu/simonlab/bioinformatics/programs/mview/manual/manual.html#colours).

Supplementary Figure 2 Sequence alignments for porcine IFNγ protein. Species orthologs of IFNγ were retrieved from the non-redundant databases of the NCBI using BLASTp searches with corresponding porcine reference amino acid sequences (NP_999113.1). All alignments were handled as noted in the legend to **Supplementary Figure 1**.

Supplementary Figure 3 Intracellular staining of pig cells with AF647 labeled anti-PoIFNγ mAbs. Frozen PBMC were cultured overnight before stimulation with BD Leukocytes activation cocktail [containing a phorbol diester (PMA), a calcium ionophore (ionomycin), and a protein transport inhibitor (Brefeldin A)]. Cells were stained with Bio-Rad viability stain and Fc receptors were blocked with rabbit serum before surface staining with αCD3 mAb. Cells were then fixed and permeabilized before intracellular staining with several αPoIFNγ mAbs. Data were collected using flow cytometry, gating on live lymphocytes and on live CD3+ T cells, and analyzed using FlowJo Software.
