# Supplementary figures and images for: Development and Characterization of New Monoclonal Antibodies Against Porcine Interleukin-17A and Interferon-Gamma
Source: Front Immunol. 2022 Feb 3;13:786396. doi: 10.3389/fimmu.2022.786396 (PMC8850701; doi:10.3389/fimmu.2022.786396)

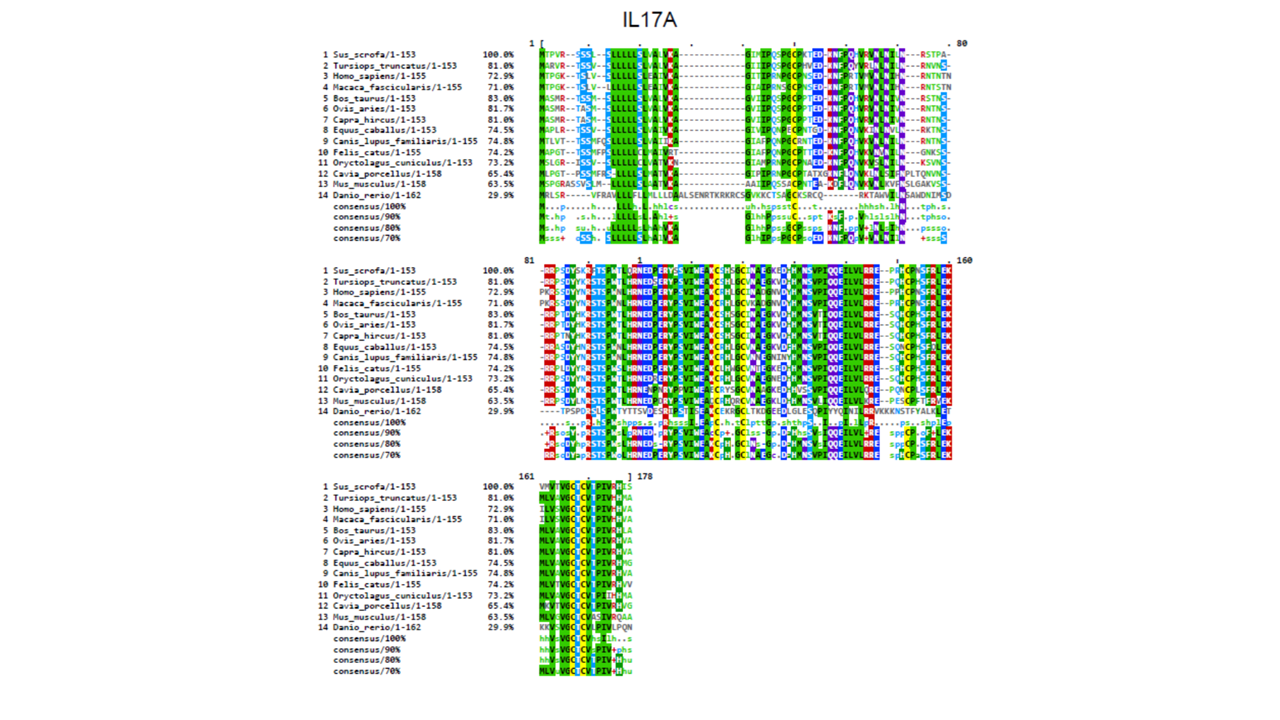

Supplement: Supplementary Figure 1 — Sequence alignments for porcine IL-17A protein. Species orthologs of IL-17A were retrieved from the non-redundant databases of the National Center for Biotechnology Information (NCBI) using BLASTp searches with the corresponding porcine reference amino acid sequences (NP_001005729.1). Retrieved sequences were aligned using the NCBI constraint-based alignment tool (COBALT). Alignment outputs, percentage identity labels and (default) consensus lines for 100%, 90%, 80% and 70% thresholds were generated using the MView tool. Consensus shading was applied using the “any” parameter. Upper case characters (amino acid residues) are shaded using the default CLUSTAL colormap. Lower case characters in consensus lines correspond to physicochemical amino acid classes (25) also available at https://vcru.wisc.edu/simonlab/bioinformatics/programs/mview/manual/manual.html#colours). [file Image_1.tif]

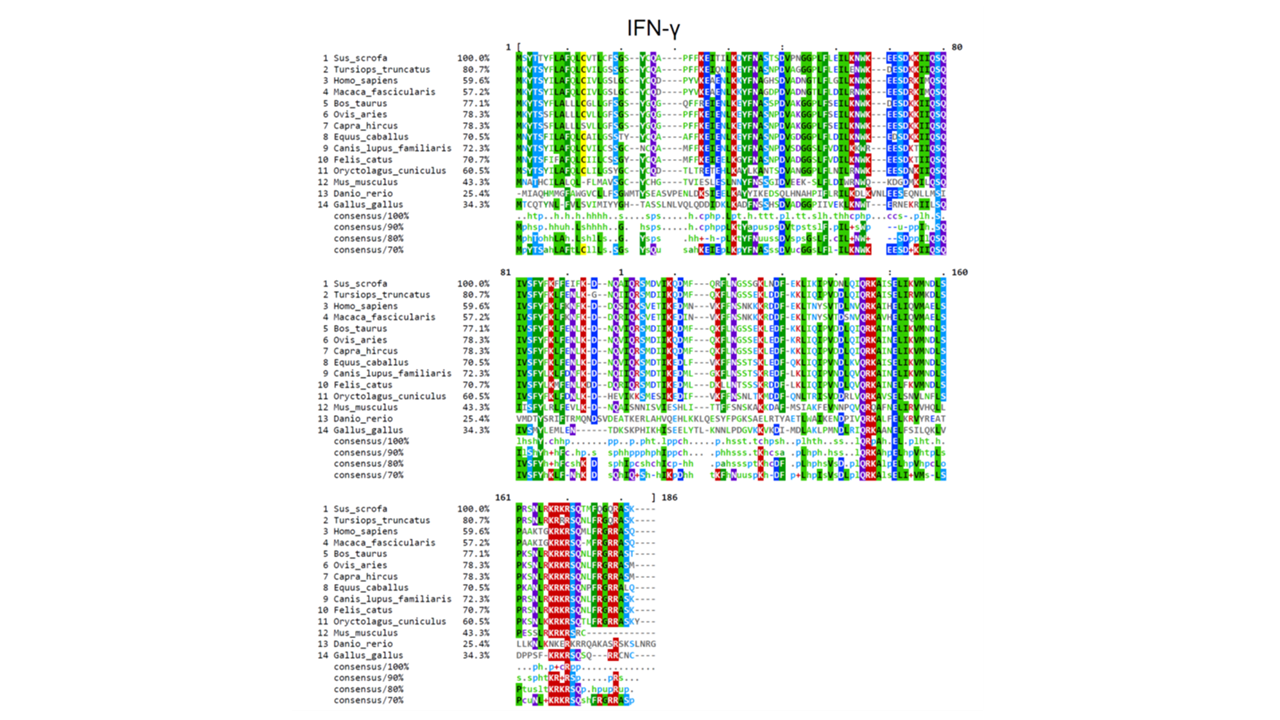

Supplement: Supplementary Figure 2 — Sequence alignments for porcine IFNγ protein. Species orthologs of IFNγ were retrieved from the non-redundant databases of the NCBI using BLASTp searches with corresponding porcine reference amino acid sequences (NP_999113.1). All alignments were handled as noted in the legend to Supplementary Figure 1 . [file Image_2.tif]

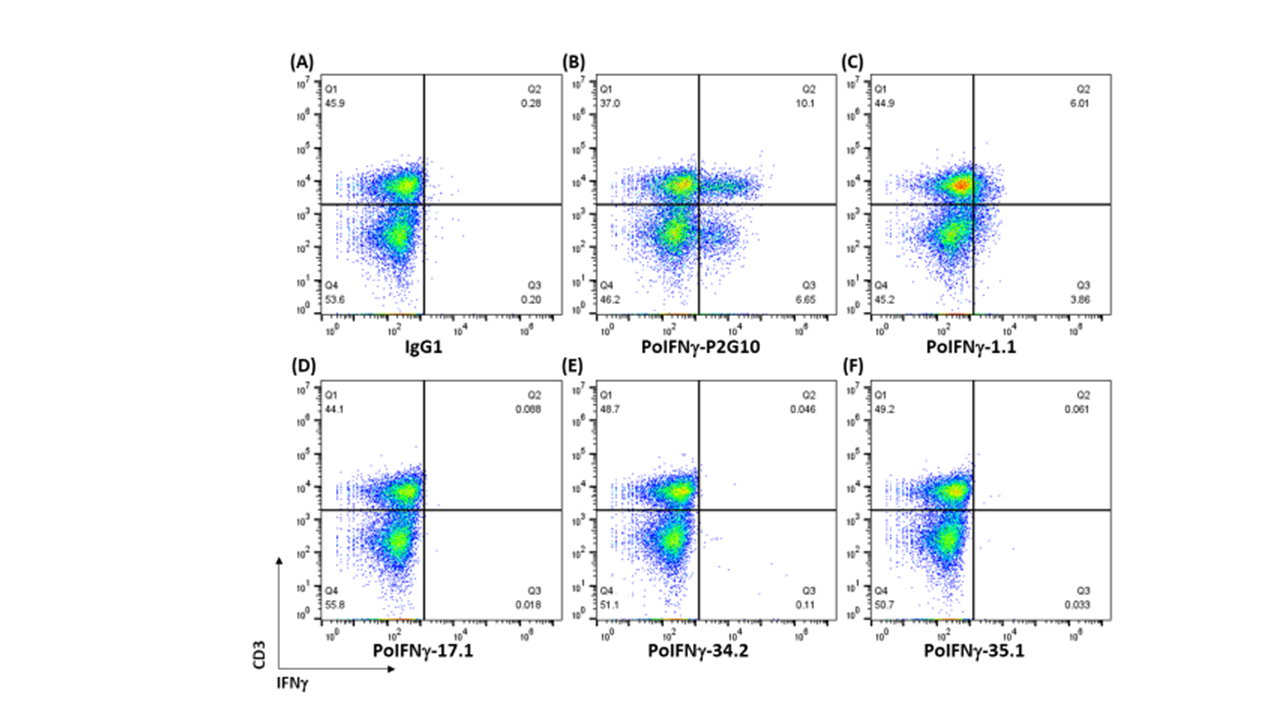

Supplement: Supplementary Figure 3 — Intracellular staining of pig cells with AF647 labeled anti-PoIFNγ mAbs. Frozen PBMC were cultured overnight before stimulation with BD Leukocytes activation cocktail [containing a phorbol diester (PMA), a calcium ionophore (ionomycin), and a protein transport inhibitor (Brefeldin A)]. Cells were stained with Bio-Rad viability stain and Fc receptors were blocked with rabbit serum before surface staining with αCD3 mAb. Cells were then fixed and permeabilized before intracellular staining with several αPoIFNγ mAbs. Data were collected using flow cytometry, gating on live lymphocytes and on live CD3+ T cells, and analyzed using FlowJo Software. [file Image_3.tif]
